# Association Between Air Pollutants and Infectious Epiglottitis Incidence: A Observational Study

**DOI:** 10.1002/hsr2.72076

**Published:** 2026-03-10

**Authors:** Pengcheng Yu, Rui Fang, Chao Xue

**Affiliations:** ^1^ ENT institute and Department of Otorhinolaryngology Eye & ENT Hospital, Fudan University Shanghai China

**Keywords:** air pollution, epiglottitis, particular matter, respiratory tract infections

## Abstract

**Background and Aims:**

Infectious epiglottitis is inflammation of epiglottis and surrounding structures and may be life‐threatening without treatment. Air pollution is a critical risk factor for human health. This study aims to explore the correlation between infectious epiglottitis and air pollutants.

**Methods:**

We collected the daily infectious epiglottitis cases in our hospital and the daily meteorological data, including average temperature, relative humidity, and the daily concentrations of air pollutants of Shanghai, China, from January, 2015 to December, 2019. Air pollutants include particulate matter (PM_2.5_ and PM_10_), sulfur dioxide (SO_2_) and nitrogen dioxide (NO_2_). Generalized additive Poisson regression models were applied to assess the association between the corresponding pollutants and hospital visits for infectious epiglottitis.

**Results:**

A total of 3280 infectious epiglottitis cases were identified with 1.80 hospital visits per day for all subjects. The mean daily concentrations of PM_2.5_, PM_10_, SO_2_, and NO_2_ were 39.7 μg/m^3^, 53.9 μg/m^3^,11.0 μg/m^3^, and 40.5 μg/m^3^, respectively. Spearman correlation analyses indicated a moderate to strong and positive correlation among air pollutants. There was a linear and increasing association between the daily concentration of PM_2.5_ and hospital visits (*p* < 0.01). The significant estimate was found in PM_2.5_ for 1‐day lag (RR = 1.041, 95% CI 1.003, 1.079). No significant association was observed among all lag days for other pollutants.

**Conclusion:**

Particulate matter 2.5, SO_2_, and NO_2_ were significantly associated with infectious epiglottitis incidence in Shanghai. The estimated hospital visits were increased by 4.1% for a 10‐μg/m^3^ increase in PM_2.5_ for 1‐day lag.

## 1 Introduction

Infectious epiglottitis refers to inflammation of the epiglottis and surrounding structures [1]. It can be life‐threatening because it leads to the supraglottic swelling which places patients at risk for airway obstruction. As a respiratory disease, infectious epiglottitis can be caused by infection and noninfectious etiologies, such as caustic digestion, thermal injury, and local trauma [2, 3, 4].

Air pollution is a critical risk factor for human health. The exposure to air pollutants can cause the epithelial and/or systemic inflammation of respiratory tract which may result in respiratory diseases such as otitis media and pneumonia [5]. Short term exposure to fine particulate pollution is associated with increased hospital admissions for major cardiovascular diseases [6]. World Health Organization estimates that in 2019, outdoor air pollution are associated with 4.2 million premature deaths worldwide [7]. Air pollution accounts for 500,000 lung cancer deaths and 1.6 million COPD deaths annually [8]. Our life was badly affected by the COVID‐19 global pandemic which has been proved to be correlated with air pollution and climatic change [9, 10, 11, 12]. Many researchers have been focusing on the association between air pollution and respiratory infections. Particulate matter (PM) air pollution is associated with an increasing risk of respiratory infections [13]. Short‐term and long‐term exposure to sulfur dioxide (SO_2_) and nitrogen dioxide (NO_2_) was significantly associated with respiratory mortality [14]. A previous study suggests that air pollutants exacerbates the airway inflammation through specific inflammatory pathways [15]. Infectious epiglottitis is a rapid and serious upper airway inflammation, and can be potentially life‐threatening due to the misdiagnosis or delayed treatment. Few studies reported the relationship between air pollutants and infectious epiglottitis incidence.

In the present study, we sought to explore the effects of weather conditions and air pollutants on infectious epiglottitis incidence by time series analysis.

## 2 Methods

### 2.1 Study Setting

This study was performed at the emergency department of Eye & ENT Hospital, Fudan University in China. Our hospital is the largest and best specialized hospital in China with nearly 1.2 million outpatient and emergency department visits per year, far exceeding the sum total of outpatient and emergency department visits in other hospitals in Shanghai. Patients receiving emergency care with a diagnosis of infectious epiglottitis in our hospital from January 1, 2015 to December 31, 2019 were included in the study. Infectious epiglottitis was diagnosed through history, clinical symptoms and physical examination. Clinical symptoms include sore throat, pain when swallowing, difficulty breathing and fever. The swelling of epiglottis could be seen in all patients. Patients with the history of caustic digestion, thermal injury, local trauma, upper airway surgeries or endotracheal intubation were excluded. We collected data from the medical records as follows: gender, age, arrival time and diagnosis. The inclusion criteria were (1) diagnosed as infectious epiglottitis and (2) first onset during an episode. This study was approved by the institutional ethics committee of our hospital (approved number: 2021182).

### 2.2 Air Pollution and Meteorological Data

Meteorological data of January 1, 2015 to December 31, 2019 were collected from the Ministry of Ecology and Environment of the People's Republic of China, China Meteorological Data Service Centre. Meteorological data includes the daily weather parameters and the daily average concentrations of air pollutants. The daily weather parameters we used in this study were average temperature (T_mean_,°C), relative humidity (RH, %), and the daily average concentrations of 4 air pollutants which included PM_2.5_ (μg/m^3^), PM_10_ (μg/m^3^), SO_2_ (μg/m^3^), and NO_2_ (μg/m^3^).

### 2.3 Statistics

First, all variables (including daily cases and meteorological parameters) were processed by descriptive analysis. Spearman correlation analyses were used to estimate the relationship among T_mean_, RH, and air pollutants.

Then, generalized additive Poisson regression models were applied to access the association between air pollutants and hospital visits for infectious epiglottitis [16]. Dependent variables in the models were daily hospital visits for infectious epiglottitis, and the independent variables were fitted by smoothing splines [17]. Weather variables (including T_mean_ and RH) were incorporate in the models according to the generalized cross‐validation (GCV) minimum principle [18]. Daily concentrations of PM_2.5_, PM_10_, SO_2_, and NO_2_ for the 0–7 day lags (lag0–lag7) were added into the model separately to analyze the lag effect of each pollutant, as shown below:

log[E(y)] = β × pollutants + s (time, df=7/year) + s (temperature, df = 4) + s (RH, df = 4) + day of the week + public holiday + *α*

where y is the daily hospital visits for infectious epiglottitis; β is the vector of coefficients for pollutants; s is the smoothing spline function; time is the date from January 1, 2015 to December 31, 2019; temperature is the daily average temperature (°C); RH is the daily average relative humidity (%); public holiday is the statutory public holiday in mainland China; *α* is the intercept.

A lag of 0 days corresponded to the association between one pollutant and the risk of hospital visits on the same day. Lag 1 or lag 2 referred to the risk of infectious epiglottitis associated with one pollutant one or 2 days before the event, respectively. The number of infectious epiglottitis cases per day was low, and we were not able to classify into more subgroups. We used the estimation of regression coefficients (β) of PM_2.5_, PM_10_, SO_2_ and NO_2_ concentrations from the model to calculate patients' risk ratios (RRs) and 95% CIs with a 10‐μg/m^3^ increase of the corresponding air pollutants. All analyses were done using R software (version 4.1.1).

## 3 Results

A total of 3280 infectious epiglottitis cases was identified in our hospital from January 1, 2015 to December 31, 2019 (Table 1). The male was diagnosed more than the female as a sex‐ratio of 1.44:1 in this study. Patients aged 40 to 60 years old accounted for nearly half of all cases (42.3%), whereas patients under 20 or over 80 years old made up for less than 2% of populations. There were 1.80 hospital visits per day for all subjects (Table 2). The daily averages for T_mean_ and RH were 17.5 ± 8.7°C and 73.6 ± 12.4% during the study period, respectively.

**Table 1:** Demographic characteristics of infectious epiglottitis cases.

	*N*	%
Total	3280	100.0
Sex		
Male	1936	59.0
Female	1344	41.0
Age		
⩾ 0, < 20	45	1.4
⩾ 20, < 40	1030	31.4
⩾ 40, < 60	1386	42.3
⩾ 60, < 80	775	23.6
⩾ 80, < 100	44	1.3

**Table 2:** Distribution of hospital visits for infectious epiglottitis and meteorological parameters, shanghai, 2015–2019.

	*N*	Min	Max	Mean	SD	Median
Total hospital visits, *N*	3280	0	10	1.8	1.5	2
Meteorological data						
Relative humidity, %	1826	28	98	73.6	12.4	75
Mean temperature,°C	1826	−6.1	34.8	17.5	8.7	18.6
Pollutants						
PM_2.5_, μg/m3	1826	5	215	39.7	27.2	32.5
PM_10_, μg/m3	1826	7	247	53.9	31.3	46
SO_2_, μg/m3	1826	4	69	11.0	6.0	10
NO_2_, μg/m3	1826	5	127	40.5	18.5	37

Abbreviations: NO_2_, (nitrogen dioxide); PM_2.5_, (particulate matter less than 2.5 μm in diameter); PM_10_, (particulate matter less than 10 μm in diameter); SO_2_, (sulfur dioxide).

Mean daily concentrations of PM_2.5_, PM_10_, SO_2_, and NO_2_ were 39.7 ± 27.2 μg/m^3^, 53.9 ± 31.3 μg/m^3^,11.0 ± 6.0 μg/m^3^, and 40.5 ± 18.5 μg/m^3^, respectively. Spearman correlation analyses indicated a moderate to strong and positive correlation among air pollutants, especially for PM_2.5_ and PM_10_ (r = 0.86, *p* < 0.01) (Table 3). There were low and negative correlations between air pollutants and weather factors.

**Table 3:** Spearman's correlation coefficients between meteorological parameters and acute epiglottitis cases.

	RH	T_mean_	PM_2.5_	PM_10_	SO_2_	NO_2_
RH	1					
T_mean_	0.12**	1				
PM_2.5_	−0.13**	−0.32**	1			
PM_10_	−0.42**	−0.30**	0.86**	1		
SO_2_	−0.37**	−0.36**	0.64**	0.69**	1	
NO_2_	−0.04**	−0.48**	0.68**	0.62**	0.50**	1

*Note:* ** Indicates significant association, significance level set at 0.01.

Abbreviations: NO_2_, (nitrogen dioxide); PM_2.5_, (particulate matter less than 2.5 μm in diameter); PM_10_, (particulate matter less than 10 μm in diameter); RH, (relative humidity); SO_2_, (sulfur dioxide); Tmean (average. temperature).

**Figure 1:**
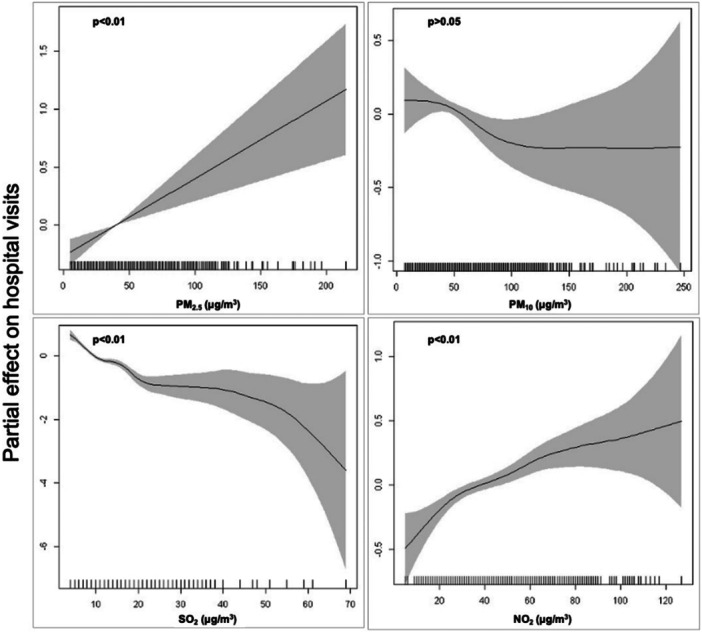
The exposure‐response relationships between the concentrations of air pollutants and hospital visits for infectious epiglottitis. The curves were estimated in generalized additive Poisson regression models using cubic regression spline with degree of freedom of 1, 3, 7, and 4 for PM_2.5_, PM_10_, SO_2_, and NO_2_, respectively.

We presented the estimated exposure‐response curves between hospital visits for infectious epiglottitis and air pollutants (Figure 1). There was a linear and increasing association between the daily concentration of PM_2.5_ and hospital visits (*p* < 0.01). We found a nonlinear and generally decreasing association between hospital visits and the daily concentration of SO_2_ (*p* < 0.01). A generally increasing association between hospital visits and the daily concentration of NO_2_ (*p* < 0.01) was found in this study. No significant association was found between the daily concentration of PM_10_ and hospital visits (*p* > 0.05).

**Figure 2:**
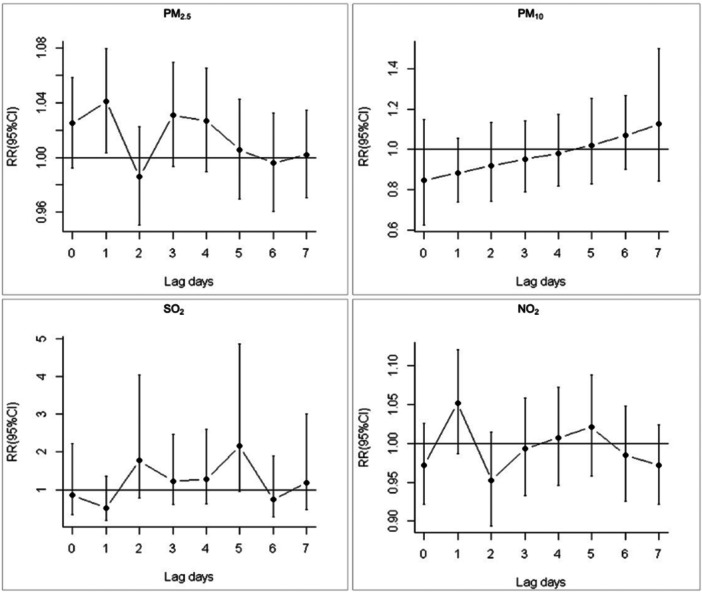
Rate ratios and 95% CIs following 10‐μg/m^3^ increases in 0–7‐day lagging air pollutants concentrations and hospital visits for infectious epiglottitis, Shanghai, 2015–2019.

Associations between infectious epiglottitis cases and a 10‐μg/m^3^ increase in lags 0–7 for the corresponding pollutants were presented (Figure 2). We calculated RRs and 95% CIs for each lag day and found highest significant estimate in PM_2.5_ for 1‐day lag (RR = 1.041, 95% CI 1.003, 1.079). No significant association was observed among all lag days for other pollutants.

## 4 Discussion

### 4.1 Mechanism of Infectious Epiglottitis

Infectious epiglottitis can be caused by bacteria, virus, fungi, and smoke inhalation [2, 3, 4]. Bacterial pathogens are the most common infectious etiology. Most of these pathogens can colonize in pharynx, which may result in infectious epiglottitis [19, 20]. However, the mechanism of the pharyngeal colonization to epiglottis is extremely complicated referring to the fluid dynamics in human airway. Meteorological factors, like air pollutants, are thought to act as a carrier of pathogens, bring these pathogens to human respiratory tract and elicit biological effects [10, 21]. There, we sought to explore the effects of weather conditions and air pollutants on infectious epiglottitis incidence. This study may, to some degree, help citizens prevent infectious epiglottitis.

### 4.2 Harm of Air Pollutants

A body of evidence suggests that climate change has great impact on the atmospheric microbiome and human respiratory health. Climate change influences the concentrations of air pollutants like PM_2.5_, PM_10_, and NO_2_ [22]. The fine particles can penetrate into the respiratory system and then cause cellular injury. Air pollutants, especially SO_2_, CO, can increase the risk of influenza‐like illness [23]. However, few studies have focused on the correlation between climate change and infectious epiglottis incidence. In this study, we found the significant association between air pollutants and infectious epiglottitis incidence.

### 4.3 Correlation Analysis

In this study, we found a male predominance for infectious epiglottitis and patients over 40 years old accounted for the highest proportion, which is consistent with previous reports [24, 25]. We assume that men are more likely exposed to smoke inhalation, which is a risk factor for infectious epiglottitis. The increasing and linear exposure‐response curve between PM_2.5_ and infectious epiglottitis cases indicated that the elevated concentration of PM_2.5_ could result in more hospital visits for infectious epiglottitis. The estimated hospital visits were increased by 4.1% for a 10‐μg/m^3^ increase in PM_2.5_ for 1‐day lag. To our knowledge, this is the first study to quantify the lagged effect of PM_2.5_ on infectious epiglottitis incidence, suggesting a 24‐h critical window for clinical intervention. Several epidemiological studies suggested that the elevated fine particles could increase the risk of respiratory infections after short‐term exposure [26, 27]. The mechanisms of the damaging effects of PM_2.5_ on the respiratory system include injury from free radical peroxidation, imbalanced intracellular calcium homeostasis and inflammatory injury [28]. The accumulation of PM_2.5_ in human upper airway might intensify the cellular and molecular damage of epiglottis, resulting in occurrence of disease. Infectious epiglottitis is primarily caused by bacterial pathogens. The pathogenesis of PM_2.5_ on infectious epiglottitis requires further investigations. Larger particles (> 8 μm) are more likely to impact upper respiratory airways due to greater inertion [29]. In this study, we found no significant association between PM_10_ and infectious epiglottitis incidence.

Sulfur dioxide is produced by burning fossil‐derived fuels and it is the main component of acid rain. The effect of SO_2_ to respiratory diseases is controversial. A multilevel meta‐analysis suggested SO_2_ showed no association with asthma exacerbations [30]. Another meta‐analysis indicated SO_2_ was significantly associated with increased cardio‐respiratory mortality risks in Chinese population [31]. The recent decrease of COVID‐19 confirmed cases in China was associated with the increase in SO_2_ [32]. In this study, the elevated SO_2_ seemed to be inverse to increased infectious epiglottitis cases according to the results of the exposure‐response curves. We assume that the acid environment caused by the combination of SO_2_ and airway secretions helps to prevent inflammation in epiglottis. But the explicit mechanism requires further study. We found no significant relative risk in SO_2_ for the 0–7 lag days. Nitrogen dioxide is another important ambient air pollutant and is mainly produced by vehicle exhaust. Long‐term exposure NO_2_ to is associated with higher mortality from respiratory disease [33]. In the present study, we found a significant increasing association between hospital visits for infectious epiglottitis and the daily concentration of NO_2_. We assumed that a long‐term exposure of NO_2_ might affect infectious epiglottitis incidence. Our results differed from a recent case‐control study. It concluded that the short‐term NO_2_ exposure were positively related to the occurrence of epiglottitis, but only patients over 40 years old were included [34].

### 4.4 Implications for Public Health

The findings highlight air pollution as additional factors influencing the incidence of infectious epiglottitis. The county government should provide effective policy guidance for accelerating the green transformation of industrial structure, optimizing waste disposal and reducing emissions for automobiles. Citizens should reduce outdoor activities and consider wearing the high‐quality, properly fitted masks outdoors when air quality is poor.

## 5 Limitations

The limitation in this study derives from the incomplete sampling because all clinical data came from only one hospital in Shanghai and meteorological data might differ from different districts of the city. Besides, we were not able to classify into more subgroups due to the small number of daily infectious epiglottitis cases. For the same reason, other variables including smoking, alcohol consumption, obesity, et al. were not considered in this study.

## 6 Conclusion

Particulate matter 2.5, SO_2_, and NO_2_ were significantly associated with infectious epiglottitis incidence in Shanghai. To prevent infectious epiglottitis, citizens may need to reduce exposure to air pollutants by staying indoors or wear face medical masks outdoors when the concentration of air pollutants rises. The estimated hospital visits were increased by 4.1% for a 10‐μg/m^3^ increase in PM_2.5_ for 1‐day lag. This means exposure to PM_2.5_ today may lead to a higher incidence of infectious epiglottitis next day. Conversely, prevention of infectious epiglottitis is feasible.

## Author Contributions

**Chao Xue:** conceptualization; supervision; writing – review and editing.

## Ethics Statement

This study was approved by the institutional review board of Eye & ENT Hospital, Fudan University (Project number: 2021182).

## Conflicts of Interest

The authors declare no conflicts of interest.

## Transparency Statement

The lead author Chao Xue affirms that this manuscript is an honest, accurate, and transparent account of the study being reported; that no important aspects of the study have been omitted; and that any discrepancies from the study as planned (and, if relevant, registered) have been explained.

## Data Availability

The data that support the findings of this study are available from the corresponding author upon reasonable request.
